# Othello Syndrome Secondary to Ropinirole: A Case Study

**DOI:** 10.1155/2012/353021

**Published:** 2012-06-24

**Authors:** Kakali Pal, Abigail Smith, Joseph Hayes, Apu Chakraborty

**Affiliations:** ^1^Highgate Mental Health Centre, Camden and Islington NHS Foundation, Dartmouth Park Hill, London N19 5NX, UK; ^2^Mental Health Sciences Unit, University College London, Charles Bell House, 67-73 Riding House Street, London W1W 7EJ, UK; ^3^Lions Gate Hospital, 231 East 15th Street, North Vancouver, BC, Canada V7L 2L7

## Abstract

This case report describes a forty-two-year-old man with no previous psychiatric history who developed delusional jealousy (Othello Syndrome) associated with ropinirole treatment. Ropinirole is a commonly used dopamine receptor agonist, which was being used to treat his Parkinson's disease, and his delusional symptoms resolved entirely with ropinirole dose reduction.

## 1. Introduction

The name Othello syndrome was first coined in 1954 [1] after the lead in Shakespeare's play *Othello*. Othello murders his wife, as he believes she has been unfaithful. The syndrome is probably inaccurately named, as it seems Othello was deceived rather than deluded about Desdemona's alleged infidelity, but the name has remained in use [2]. Perhaps more appropriately termed morbid or delusional jealousy, the presentation is rare in its pure form and is more commonly associated with personality disorder, chronic mental illness, substance misuse, and organic brain lesions [3]. Clinicians must, furthermore, be aware of the significant risk of attempted suicide and homicide (20% and 17% resp. in a UK sample) [4].

Ropinirole is a commonly used dopamine receptor agonist with a high affinity for D2 subfamily receptors, in particular the D3 subtype. It is licensed for use in Parkinson's disease and Restless Leg Syndrome in the UK.

The case presented below describes a forty-two-year-old gentleman with no previous psychiatric history who developed delusional jealousy (Othello syndrome) associated with the use of ropiniorole.

## 2. Case

A forty-two-year-old Kosovan man presented with a two-week history of delusional beliefs that his wife was having an affair. He had been diagnosed with Parkinson's disease three years previously, but had no past psychiatric history.

His presentation was consistent with a diagnosis of Othello syndrome, which is characterised by intense delusional beliefs of infidelity by the patient's spouse or sexual partner [3]. In this case the patient developed delusions of reference, which led him to believe that his wife was being unfaithful; he reported that images of fruit displayed on his wife's social network page signalled that she was having an affair.

As a result, he began placing fruit around their house in order to demonstrate his discovery. As the delusions intensified he developed feelings of shame, which led him to brandish a knife and threaten to cut his wrists. This resulted in his admission to an acute psychiatric ward.

There was no significant affective component and no disturbance in biological function, nor did he describe ongoing suicidal ideation. He expressed delusions of reference and jealousy, but there was no evidence of thought disorder, abnormal perceptions, or cognitive impairment. He had not been misusing alcohol or drugs and did not smoke. There were no preceding personality or behavioural changes, which are rare recognised side effects of ropinirole use (such as gambling, hypersexuality, binging, or other compulsive acts) [5].

He had no significant past medical history apart from Parkinson's disease. This was being managed with ropinirole (8 milligrams, three times daily) and rasagiline (1 milligram, once daily). The ropinirole dose was last increased four months prior to his presentation, from 6 milligrams three times daily. Although he was concordant with the medication, he had started to take all his medication together at night (24 milligrams of ropinirole and 1 mg rasagiline).

He had no psychiatric or medical family history. His developmental history was unremarkable. There was no suggestion of any interpersonal or social difficulties that could have contributed to the onset of the above symptoms, including no indication of marital difficulties. He was self employed and managed his own restaurant. Routine examination revealed mild signs of Parkinsonism only. Blood tests and neuroimaging were unremarkable and did not explain his psychiatric presentation.

Rather than introducing antipsychotic medication at this stage, his ropinirole dose was reduced to 6 milligrams three times a day at appropriate intervals. His mental state subsequently improved over a period of six weeks, and a complete resolution of symptoms allowed discharge back to community. He remained well, with no reemergence of psychotic symptoms.

## 3. Discussion

The lack of predisposing factors for a psychotic disorder in this case, combined with the clear resolution of the episode, with only a reduction in ropinirole, strongly indicates a causal relationship between the use of the dopamine agonist and psychotic symptoms.

There is a recognised relationship between dopamine function and psychosis illustrated by the strong antipsychotic effects of dopamine antagonists. It is therefore conceivable that as a D2 receptor agonist, ropinirole may induce psychosis. However, in theory, ropinirole should be less likely to cause psychotic symptoms than other dopamine agonists, as it has a high affinity for D3 receptors, and should not excessively stimulate D2 receptors in the mesolimbic pathway [6].

Cases of psychosis with use of ropinirole have been described in patients who have an underlying vulnerability to developing mental illness [7]. A study investigating the relationship between dopaminergic treatment and development of Othello syndrome in patients with Parkinson's disease reported cases where an antipsychotic medication was used as treatment, as well as reducing the dose of dopaminergic agent [8]. The case above adds to the evidence for the occurrence of ropinirole induced psychosis, as the psychotic symptoms resolved with reduction of dopaminergic treatment only, suggesting that ropinirole was the root cause of the psychosis.

## 4. Conclusion

Whilst it is established that dopamine agonists should be used with caution in patients with a predisposition to developing psychiatric disorders, this case suggests that patients treated with ropinirole, who do not have an identified vulnerability, are also at risk of developing psychotic symptoms. Clinicians should be aware of this possible side effect and monitor for any changes in mental state.
